# Type 1 diabetes stigma: A UK survey of adults living with type 1 diabetes

**DOI:** 10.1111/dme.70365

**Published:** 2026-05-18

**Authors:** Harriet Housby, Akaal Kaur, Ian Godsland, Thomas Wylie, Nick Oliver, Katrina Scior, Vicky McKechnie

**Affiliations:** ^1^ Department of Metabolism, Digestion and Reproduction Imperial College London London UK; ^2^ Research Department of Clinical, Educational and Health Psychology University College London London UK

**Keywords:** social stigma, stigma, type 1 diabetes

## Abstract

**Aims:**

Type 1 diabetes stigma (T1D‐stigma) refers to negative social judgement towards people living with type 1 diabetes (T1D) and is linked to poorer psychological well‐being and suboptimal diabetes self management. This observational study aimed to explore sociodemographic, diabetes health and well‐being factors linked to high stigma scores to inform future, targeted intervention studies.

**Methods:**

UK‐based adults (aged ≥18 years) with T1D completed an online survey comprising sociodemographic and diabetes health questions, wellbeing measures for anxiety (Generalised Anxiety Disorder Scale: GAD‐7), diabetes impact (Impact of Diabetes Profile: DIDP) and depression (Patient Health Questionnaire: PHQ‐9) and the Type 1 Diabetes Stigma Assessment Scale (DSAS‐1). Potential relationships between sociodemographic, diabetes health and well‐being factors and DSAS‐1 score were identified by univariate regression and the independence of significant (*p* < 0.05) relationships was explored in multivariable regression.

**Results:**

Of 438 participants, 96% endorsed one or more items on the DSAS‐1. In multivariable regression, age (*β* = −0.136, *p* = 0.006), sex (*β* = 0.114, *p* = 0.006), BMI (*β* = 0.112, *p* = 0.023), insulin pump use (*β* = 0.108, *p* = 0.015), DIDP score (*β* = 0.211, *p* = <0.001), GAD‐7 score (*β* = 0.192, *p* = 0.002) and PHQ‐9 score (*β* = 0.195, *p* = 0.002) predicted DSAS‐1 score. Ethnicity did not predict DSAS‐1 score, nor did age at diagnosis or number of severe hypoglycaemia episodes.

**Conclusion:**

Most participants reported at least one instance of T1D‐stigma. High DSAS‐1 scores in the United Kingdom were predicted by younger age, being a woman, higher BMI, insulin pump use, higher diabetes impact and anxiety and depression scores. Future studies should further explore the impact of T1D‐stigma within these groups to tailor appropriate interventions.

**What's new?**

**What is already known?**

- Type 1 Diabetes Stigma (T1D‐stigma) is a pervasive issue that impacts psychosocial well‐being and diabetes management for people living with type 1 diabetes.

**What this study has found**

- Most participants reported at least one instance of T1D‐stigma. High DSAS‐1 scores in the United Kingdom were predicted by younger age, being a woman, higher BMI, insulin pump use, higher diabetes impact and anxiety and depression scores.

**What are the implications of the study?**

- Healthcare providers should be mindful that stigma, particularly feeling blamed and judged by others, is a common experience and may negatively affect type 1 diabetes self management.
- Future studies should further investigate the impact of T1D‐stigma within these groups to inform the development of tailored interventions.

## 1 INTRODUCTION

Type 1 diabetes (T1D) is an autoimmune condition that requires continuous and often visible self management, including glucose monitoring, insulin administration and carbohydrate counting to prevent hypoglycaemia and hyperglycaemia, which can result in serious short and long‐term complications.^1^ This complex self management often occurs within social contexts that lack awareness of, or accommodation for, the condition.^2^, ^3^, ^4^ The management of T1D, therefore, involves more than medical care; it also requires the navigation of social interactions that may carry spoken and unspoken biases or misconceptions.^5^

Recently, increased attention has been directed toward the role of health stigma in shaping the experiences of those living with long‐term health conditions, with evidence showing its impact on mental health, treatment adherence and healthcare engagement.^6^ T1D‐stigma encapsulates a range of negative social judgements, including blame for the condition, criticism of self management, differential treatment, or reduction of the person's identity to their diagnosis.^7^, ^8^ While a review of studies which looked at the attitudes of those without diabetes suggests that the public does not view diabetes as a condition for which people would be stigmatised,^3^, ^9^, ^10^ studies that involve people with lived experience of T1D consistently report that a majority have experienced what they would describe as T1D‐stigma directly.^7^ National surveys using quantitative Patient Reported Outcome Measures (PROMs) find that this is the case in Switzerland,^11^ Denmark,^12^ the United States,^13^ Canada^4^ and Australia,^14^ demonstrating the widespread experience of T1D‐stigma and associations with poorer psychological wellbeing^3^, ^15^ and suboptimal diabetes management.^2^ This discrepancy between public perception and the lived experiences of those with T1D suggests a lack of awareness regarding the types of interactions and attitudes that are experienced as stigmatising by people with T1D.

Although large‐scale surveys measuring T1D‐stigma prevalence using validated tools have not yet been conducted in the United Kingdom, stigma has been cited as having a significant impact on life with T1D in this country, and a priority for research by those living with the condition.^16^ The recent international consensus on diabetes stigma recommends assessment of the experiences and extent of diabetes stigma across countries.^3^ Some contributing and potentially mitigating factors have been identified, including the use of stigmatising language in diabetes care,^17^ for which guidelines now exist,^18^ and individual‐level traits such as self‐esteem.^14^ However, little is known about the perceived and experienced stigma encountered by adults living with T1D in the United Kingdom, including how it differentially affects subgroups within this population. Moreover, published pilot data for the United Kingdom that could inform robustly designed observational and intervention studies barely feature in the published literature.

Our observational study, therefore, aims to address these gaps by investigating demographic, clinical and psychosocial factors associated with higher stigma scores among adults living with T1D in the United Kingdom. In doing so, it seeks to highlight populations most vulnerable to stigma and lay the groundwork for tailored interventions that can mitigate its impact and ultimately improve both psychological well‐being and diabetes management for individuals living with T1D.

## 2 METHODS

### 2.1 Participants and procedure

A national, cross‐sectional survey was conducted between August 2024 and April 2025 to explore stigma experiences of adults living with T1D in the United Kingdom. Eligibility criteria included being aged 18 or older, living in the United Kingdom with T1D, and having the ability to complete the survey online or by post in English.

Participants were recruited through three primary pathways. First, invitations were sent via text message, email, phone call, or post to *N* = 3754 individuals identified through the After Diabetes Diagnosis REsearch Support System (ADDRESS‐2). ADDRESS‐2 is a national study, active since 2011, that individuals newly diagnosed with T1D can join to receive information about relevant research opportunities. ADDRESS‐2 participants are relatively representative of the broader national T1D population. Comparisons with the National Diabetes Audit prevalence data show similar proportions of males (56% vs. 56% in ADDRESS‐2 and NDA, respectively) and some difference in White ethnicity (89% vs. 85%, respectively). Second, *N* = 463 individuals who had previously participated in T1D research at Imperial College London and had consented to be contacted about future studies were invited via email. Third, the study was promoted on social media platforms. The study partnered with Egality Health, a community engagement agency, to develop tailored recruitment materials aimed at engaging groups historically under‐represented in research. Egality Health also helped disseminate these materials through community organisations with which they are affiliated.

For those participants who were directly invited to participate in the study, invitation materials (email, letter or phone call according to stated preference) explicitly stated that this was a survey study about T1D‐stigma. Materials produced by Egality Health for the third recruitment stream also explicitly referenced diabetes stigma and were co‐produced with community leaders. These materials included videos of people from minoritised ethnicities discussing some of their experiences of T1D‐stigma, as well as advertisements for social media, some of which explicitly stated that the researchers wanted to hear from people who were from ‘diverse ethnic communities’.

The online survey was hosted by Qualtrics™, beginning with a plain English information sheet and a series of eligibility screening questions, followed by a consent form and the survey. Participants also had the option to complete the survey via post and a prepaid return envelope.

Ethical approval was obtained through Imperial College Research Ethics Committee (ICREC). ICREC Reference number 6986692.

### 2.2 Measures

#### 2.2.1 Sociodemographic and diabetes health

A series of sociodemographic and diabetes health‐related characteristics were self reported by participants.

#### 2.2.2 Type 1 Diabetes Stigma Assessment Scale‐1 (DSAS‐1)

The DSAS‐1^8^ is a 19‐item scale assessing perceived and experienced stigma in individuals with T1D across three subscales: Treated Differently (six items), Identity Concerns (seven items) and Blame and Judgement (six items). Items are rated on a 5‐point Likert scale (1 = strongly disagree to 5 = strongly agree), with higher scores indicating greater perceived or experienced stigma. Total scores range from 19 to 95; subscale ranges are 6–30 (Treated Differently), 7–35 (Identity Concerns) and 6–30 (Blame and Judgement). All subscales showed high internal consistency (Cronbach's *α* = 0.88–0.89) and the scale demonstrated satisfactory concurrent, convergent and discriminant validity.^8^ An open‐text question inviting participants to expand on their experiences with T1D‐stigma was also added for participants who responded ‘Agree’ (4) or ‘Strongly Agree’ (5) to any of the items within the measure. A thematic analysis of open‐text responses is presented elsewhere.^19^

#### 2.2.3 Impact of Diabetes Profile (DIDP)

The DIDP^20^ was designed as a brief assessment of the perceived impact of diabetes on seven key dimensions of life: physical health; finances; relationships; leisure activities; work or studies; emotional wellbeing; dietary freedom. Each item uses a seven‐point Likert scale (1 = very positive impact, 7 = very negative impact). A ‘not applicable’ (N/A) response option is also available for each item. Total measure scores range from 0 to 49. Higher scores indicate a greater negative impact of diabetes across global life dimensions. The scale has high internal consistency (*α* = 0.85–0.90) and satisfactory convergent and discriminant validity.^20^

#### 2.2.4 Generalised Anxiety Disorder 7 (GAD‐7)

The GAD‐7^21^ is a screening tool for identifying probable cases of generalised anxiety disorder. It is a seven‐item three‐point Likert scale where each item is scored from 0 (not at all) to 3 (nearly every day). Total scores range from 0 to 21, with scores of 5, 10 and 15 representing cut‐offs for mild, moderate and severe levels of anxiety. The scale has good convergent validity and good test–retest reliability (intraclass correlation = 0.83).^21^

#### 2.2.5 Patient Health Questionnaire‐9 (PHQ‐9)

The PHQ‐9^22^ is the depression module of the wider, self‐administered Patient Health Questionnaire for common mental disorders. Each of the nine items corresponds with the nine DSM‐IV criteria for depression and is scored 0 (not at all) to 3 (nearly every day). PHQ‐9 scores range from 0 to 27, with scores of 5, 10, 15 and 20 representing cut‐offs for mild, moderate, moderately severe and severe depression. The scale has strong evidence for its validity as a brief measure of depression severity, including criterion, construct and external validity.^22^

### 2.3 Data analysis

Descriptive self‐reported data on sociodemographic and clinical characteristics are presented as counts and percentages for the categorical variables, and means/medians and standard deviations (SD)/interquartile ranges (IQR) are presented for the continuous variables.

Mean differences in diabetes stigma (using total DSAS‐1 score) were tested through independent sample *t*‐tests or one‐way ANOVA when the variables had more than two categories. Tukey HSD post‐hoc test was used to analyse pairwise differences. Correlations between continuous variables were calculated with Pearson's correlation (*r*).

Potentially independent associations with DSAS‐1 score were identified in univariate linear regression analyses, which were performed on continuous and binary categorical data. Variables that significantly correlated with total DSAS‐1 scores at *p* < 0.05 were assessed for multicollinearity and then entered into multivariable regression analyses to identify independent associations. All predictors were entered into the regression model simultaneously to adjust for each other. Analyses were performed using the IBM Statistical Package for the Social Sciences (SPSS) Statistics for Windows, version 29.0. Statistical significance was defined as *p* < 0.05.

## 3 RESULTS

Overall, 380 of the 4217 invited participated (9% response rate), with *N* = 253/3754 recruited from ADDRESS‐2 and *N* = 127/463 from Imperial College London. An additional *N* = 58 were recruited via social media advertisements, producing a sample of *N* = 438. The median age was 42.0 years (IQR 31.0–55.0), the majority of participants were White (91.8%; *N* = 402), women (61.0%; *N* = 266), university educated (70.3%; *N* = 308) and lived in less deprived postcodes (IMD_Mdn_ = 7; IQR = 4–9). The median body mass index (BMI) was 25.0 kg/m^2^ (IQR = 22.6–28.6). The median age of diabetes diagnosis was 25.0 years (IQR 14.0–39.0), with a median diabetes duration of 10.0 years (IQR = 7.0–24.5). The median HbA1c was 52 mmol/mol (IQR = 46–58), with time in range of 74% (IQR = 60–85). The ADDRESS‐2 study team provided summary statistics on those who consented and those who did not based on gender and ethnicity. Women were more likely to respond to being contacted than men (15% vs. 10%, *p* < 0.001), but there was no difference in the proportions of people from non‐White ethnicities that did not respond compared to those that did (13.6% vs. 12.4%, *p* = 0.617). Further sociodemographic, clinical and psychosocial information is provided in Table 1.

**TABLE 1:** Sociodemographic, clinical and psychosocial characteristics of participants (*N* = 438).

Participant characteristics		
Sociodemographic characteristics		
Women, *N* (%)	266	(61)
Age, years (Mdn, IQR)	42.0	(31.0–55.0)
Ethnicity, *N* (%)		
White	402	(92)
Black	16	(3.6)
Mixed	14	(3.1)
Asian	6.0	(1.3)
Religion (*N*, %)		
No religion	168	(38)
Christian	159	(36)
Atheist	53	(12)
Agnostic	23	(5.3)
Prefer not to say	19	(4.3)
Other	16	(3.7)
Education (*N*, %)		
Undergraduate degree	158	(36)
Postgraduate degree	150	(34)
A‐level	72	(16)
GCSE or equivalent	37	(8.4)
Apprenticeship	16	(3.7)
Other	5.0	(1.1)
IMD	7	(4–9)
Clinical characteristics		
Age diagnosed with diabetes (years) (Mdn, IQR)	25.0	(14.0–39.0)
BMI	25.0	(22.6–28.6)
Diabetes duration (years) (Mdn, IQR)	10.0	(7.0–24.5)
HbA1c (mmol/mol) (Mdn, IQR)	52	(46–58)
Time in range (%) (Mdn, IQR)	74	(60–85)
Number of hypos in the last week (Mdn, IQR)	2	(1–4)
Number of severe hypos in last year (Mdn, IQR)	0	(0–0)
Insulin delivery (*N*, %)		
Multiple daily injections	238	(54)
Insulin pump with CGM linkage	145	(33)
Insulin pump	55	(13)
Glucose monitoring (*N*, %)		
Continuous glucose monitoring	411	(94)
Self monitoring of blood glucose	27	(6.0)
Diabetes course attendance (*N*, %)	269	(61)
Retinal screening past year (*N*, %)	422	(96)
Urine sample past year (*N*, %)	363	(83)
Foot check past year (*N*, %)	361	(82)
Psychosocial characteristics		
Diabetes impact (DIDP; 0–49) (Mdn, IQR)	34.0	31–37
Anxiety (GAD‐7; 0–21) (Mdn, IQR)	5.0	2.0–9.0
Depression (PHQ‐9; 0–24) (Mdn, IQR)	5.0	2.0–9.0
Diabetes stigma (*M*, SD)		
DSAS‐1—total score (19–95)	56.7	14.5
Blame and judgment (7–35)	21.5	5.4
Identity concerns (6–30)	20.7	6.6
Treated differently (6–30)	14.5	5.1

Abbreviations: CGM, continuous glucose monitors; DIDP, Impact of Diabetes Profile; DSAS‐1, Type 1 Diabetes Stigma Assessment Scale; GAD‐7, Generalised Anxiety Disorder Scale; IQR, interquartile ranges; PHQ‐9, Patient Health Questionnaire‐9.

### 3.1 DSAS‐1

The mean DSAS‐1 total score was 56.6 (SD 14.4) with 96% (*N* = 420) of the sample endorsing one or more items. The majority of participants (92%, *N* = 401) endorsed one or more items on the Blame and Judgement subscale, 82% endorsed one or more items on the Identity Concerns subscale, and 56% on the Treated Differently subscale. Table 2 shows the percentage agreement with each item within the three subscales, with frequencies in the Appendix A.

**TABLE 2:** The percentage of respondents agreeing/strongly agreeing with each of the 19 DSAS‐1 items. *N* = 438.

Subscale and item wording (item number)	Agreement level	
	*n*	%
Blame and Judgement		
Some people make unfair assumptions about what I can and cannot do because of my type 1 diabetes (1)	335	77
Because I have type 1 diabetes, some people judge me if I eat sugary food or drinks (e.g., cakes, lollies, soft drink) (14)	317	73
Some people assume that it is my fault I have type 1 diabetes (e.g., I ate too much sugar, I could have prevented it) (9)	299	68
Some people think I'm irresponsible when my diabetes management isn't ‘perfect’ (4)	254	58
Some people think I need insulin because I haven't looked after myself (11)	248	57
Some people think that I brought type 1 diabetes on myself (17)	247	56
Identity concerns		
I feel embarrassed about what people might think if I need help with a hypo (5)	260	59
I worry what people will think if they see me injecting/bolusing insulin or checking my blood glucose in public (13)	201	46
I feel self‐conscious about all the tools I need to manage my type 1 diabetes (e.g., insulin pen, pump, blood glucose meter) (7)	198	45
If I were to inject insulin in public, people would think I was taking drugs (16)	198	45
I feel embarrassed when I have to manage my type 1 diabetes in public (e.g., check blood glucose, inject/bolus insulin, refuse food, eat extra food) (10)	196	45
I feel worried about telling people I have type 1 diabetes in case they react negatively (18)	145	33
To avoid negative reactions, I don't tell people I have type 1 diabetes (2)	136	31
Treated differently		
Some people expect less of me because I have type 1 diabetes (19)	117	27
Some people see me as a lesser person because I have type 1 diabetes (3)	115	26
I have been discriminated against in the workplace because I have type 1 diabetes (6)	109	25
Some people think I'm unreliable because I have type 1 diabetes (15)	90	21
I have been rejected by others (e.g., friends, colleagues, romantic partners) because of my type 1 diabetes (12)	67	15
Because I have type 1 diabetes, I have been excluded by others from certain social events (8)	57	13

*Note*: Individual item agreement = ‘4’ agree or ‘5’ strongly agree. Total subscale (≥1 item agree or strongly agree).

Abbreviation: DSAS‐1, Type 1 Diabetes Stigma Assessment Scale.

The extent to which participants endorsed the stigma statements ranged from 13% for the statement ‘Because I have type 1 diabetes, I have been excluded by others from certain social events’ from the Treated Differently subscale to 76.5% for the statement ‘Some people make unfair assumptions about what I can and cannot do because of my type 1 diabetes’ from the Blame and Judgement subscale.

### 3.2 Diabetes stigma according to sociodemographic, clinical, psychological and behavioural characteristics

Table 3 presents mean differences in, or associations with, diabetes stigma (total DSAS‐1 score) according to participants' sociodemographic, clinical, and well‐being measures.

**TABLE 3:** Mean differences in, or associations with, diabetes stigma (total DSAS‐1 score) according to participants' sociodemographic, clinical and psychosocial measures (*N* = 438).

	Diabetes stigma	
	*r*	*p*
Sociodemographic		
Age	−0.266	<0.001
BMI	0.180	<0.001
IMD	−0.054	0.293
Clinical		
Age diagnosed with diabetes (years)	−0.181	<0.001
Diabetes duration (years)	−0.086	0.071
HbA1c (mmol/mol)	0.063	0.271
Time in range (%)	−0.054	0.290
Number of hypos in the last week	0.012	0.817
Number of severe hypos in the last year	0.153	0.001
Psychosocial measures		
Diabetes impact (DIDP; 0–49)	0.410	<0.001
Anxiety (GAD‐7; 0–21)	0.499	<0.001
Depression (PHQ‐9; 0–24)	0.521	<0.001

	*M* (SD)	*p*
Sociodemographic		
Sex		
Women	59.17 (13.93)	<0.001
Men	52.74 (14.56)	
Ethnicity		
White	56.00 (14.19)	<0.001
Ethnically minoritised	63.89 (16.21)	
Religion		
Religion	56.66 (14.78)	0.488
No religion	56.62 (14.11)	
Education Level		
University	56.68 (14.90)	0.468
Did not attend university	56.56 (14.36)	
Clinical		
Insulin delivery		
Multiple daily injections	54.47 (14.41)	0.002
Insulin pump with CGM linkage	58.61 (13.99)	
Insulin pump	60.87 (14.84)	
Glucose monitoring		
Continuous glucose monitoring	56.55 (14.22)	0.290
Self monitoring of blood glucose	58.15 (18.59)	
Diabetes course attendance		
Yes	56.24 (14.13)	0.229
No	57.30 (15.12)	
Retinopathy check in the last year		
Yes	56.62 (14.56)	0.412
No	57.44 (13.42)	
Urine sample from last year		
Yes	56.53 (14.41)	0.362
No	57.19 (15.08)	
Foot check in the last year		
Yes	56.08 (14.51)	0.079
No	59.29 (14.29)	

Abbreviations: BMI, body mass index; CGM, continuous glucose monitors; DIDP, Impact of Diabetes Profile; DSAS‐1, Type 1 Diabetes Stigma Assessment Scale; GAD‐7, Generalised Anxiety Disorder Scale; PHQ‐9, Patient Health Questionnaire‐9.

#### 3.2.1 Sociodemographic factors

There was a weak negative correlation between age and DSAS‐1 score (*r* = −0.266, *p* < 0.001) and a weak positive correlation between BMI and DSAS‐1 score (*r* = 0.180, *p* < 0.001). Women scored higher on the DSAS‐1 compared to men, with a mean score of 59 (SD = 13.9) versus 53 (SD = 14.6) (*p* < 0.001). Participants from White backgrounds scored lower on the DSAS‐1 than those from ethnically minoritised backgrounds, with a mean score of 56 (SD = 14.2) versus 64 (SD = 16.2) (*p* < 0.001). There was no relationship between IMD and DSAS‐1 score, and no difference between education level, nor between those reporting a religion versus those reporting no religion.

#### 3.2.2 Diabetes health

There was a weak negative correlation between age of diagnosis and DSAS‐1 score (*r* = −0.181, *p* < 0.001) and also between the number of severe hypoglycaemic episodes experienced in the last year and DSAS‐1 score (*r* = 0.153, *p* = 0.001).

A one‐way ANOVA revealed a difference in DSAS‐1 score between insulin delivery methods (*p* = 0.002). Post hoc Tukey tests showed that DSAS‐1 scores from participants using an insulin pump (mean [SD] 60.9 [14.8]) and those using an insulin pump with CGM linkage (58.6 [14.0]) were significantly higher than those using multiple daily injections (MDI) (54.47 [14.4]; *p* = 0.008, *p* = 0.017 respectively), but no difference was found in DSAS‐1 scores between using an insulin pump and insulin pump with continuous glucose monitor (CGM) linkage (*p* = 0.580).

There was no relationship between DSAS‐1 score and diabetes duration, HbA1c, time in range or number of hypoglycaemic episodes experienced in the last week. There was no difference between glucose monitoring method (blood self monitoring vs. CGM), diabetes course attendance (yes/no), retinopathy appointment attendance (yes/no), foot check attendance or submitting a urine sample (yes/no) and DSAS‐1 scores.

#### 3.2.3 Psychosocial measures

There was a strong positive correlation between diabetes impact (*r* = 0.410, *p* < 0.001), anxiety scores (*r* = 0.499, *p* < 0.001), depression scores (*r* = 0.521, *p* < 0.001) and DSAS‐1 scores. Table 3 shows mean differences in, or associations with, diabetes stigma (total DSAS‐1 score) according to participants' demographic, clinical and psychosocial measures.

### 3.3 Multivariable regression

Multivariable regression suggested that the variance in DSAS‐1 score was independently explained by younger age (*β =* −0.136, *p* = 0.006), female sex (*β =* 0.114, *p* = 0.006), higher BMI (*β =* 0.095, *p* = 0.023), insulin pump use (*β =* 0.108, *p* = 0.015), diabetes impact (*β =* 0.211, *p* = 0.001), anxiety (*β =* 0.192, *p* = 0.002) and depression (*β =* 0.195, *p* = 0.002) scores. Ethnicity did not predict DSAS‐1 score, nor did age at diagnosis or number of severe hypoglycaemia episodes. Table 4 shows the regression coefficients for these variables.

**TABLE 4:** Multiple variable analysis between DSAS‐1 total score and demographic, clinical and psychosocial variables brought forward from previous significant linear regressions (*N* = 438).

Predictors	*β* [95% CI]	*p*
Age	−0.136 [−0.213, −0.037]	0.006
Sex	0.114 [0.966, 5.655]	0.006
Ethnicity	0.057 [−1.277, 7.516]	0.164
BMI	0.095 [0.035, 0.475]	0.023
Age diagnosed	0.001 [−0.870, 0.088]	0.992
Severe hypo	0.055 [−0.760, 0.380]	0.190
Insulin delivery	0.108 [0.615, 5.591]	0.015
Anxiety	0.192 [0.194, 0.864]	0.002
Diabetes impact	0.211 [0.336, 0.807]	<0.001
Depression	0.195 [0.178, 0.804]	0.002

*Note*: Predictors were brought forward from previous significant linear regressions and entered into the model simultaneously.

Abbreviations: BMI, body mass index; CI, confidence interval; DSAS‐1, Type 1 Diabetes Stigma Assessment Scale.

## 4 DISCUSSION

The results of this United Kingdom‐based survey of adults living with T1D provide insight into different sociodemographic factors and clinical characteristics associated with experiencing T1D‐stigma.

In line with studies in other countries,^1^, ^11^, ^12^, ^13^, ^14^ the majority of the sample reported experiencing at least one form of T1D‐stigma, highlighting that T1D‐stigma is also a pervasive issue in the United Kingdom. In line with similar studies in other countries, this study did not manage to recruit a representative sample of people living with T1D, and this is considered further in the limitations section below.

The highest and lowest item endorsements, relating to unfair assumptions and being excluded from social events, match those in a multi‐study cross‐country examination of T1D‐stigma.^23^ Similarly, our findings support that ‘blame and judgement’ items were rated more highly overall than items in other subscales, followed by ‘identity concerns’ and then ‘being treated differently’.^23^ However, it is worth noting that the mean scores in our study for each subscale are consistently higher than those reported in the multi‐study cross‐country examination, perhaps indicating that our UK sample perceives or experiences more stigma.^23^

Notably, higher levels of perceived or experienced T1D‐stigma, as measured by DSAS‐1, were weakly associated with several demographic and clinical factors. Participants who scored highly on the DSAS‐1 tended to be women, younger adults, insulin pump users, have a higher BMI and self‐report symptoms of anxiety, depression and being highly impacted by their diabetes in day‐to‐day life.

The findings that women are more likely to report higher stigma scores align with other published data,^1^ which are consistent with broader gender‐based healthcare literature, highlighting the challenges women face due to sex‐based discrimination.^24^ A review of diabetes management in India found that girls are particularly at risk of T1D‐related discrimination.^25^ Similarly, a European‐wide survey of people living with T1D found that women are more likely to report experiencing depression.^26^ These findings suggest that there are unique challenges for women that may exacerbate their experiences of T1D‐stigma.^1^

Our study also supports findings that younger adults report experiencing higher levels of T1D‐stigma.^1^ Younger adults may be more vulnerable to stigma due to heightened concerns about social acceptance and identity formation during early adulthood.^1^ This aligns with a review of identity development research in young people living with T1D, highlighting the complex nature of navigating adolescence and young adulthood whilst living with T1D.^27^ In a study of fourteen 20–34‐year‐olds with T1D, young adults reported experiencing high levels of stigma, which often results in delayed management, anger and distress, suggesting that young adults require strategies to cope with T1D‐stigma and its impact.^28^

Interestingly, insulin pump users reported greater stigma than those using MDI. While insulin pump therapy is often associated with improved quality of life, including a reduction in disabling hypoglycaemic episodes, and the potentially stigmatised associated behaviours^29^, ^30^ it may also act as a visible marker of disease, drawing unwanted attention and reinforcing perceptions of difference.^31^, ^32^ This finding supports previous research indicating that the visibility of diabetes technology can sometimes heighten feelings of self‐consciousness.^13^, ^33^ However, whilst the number of participants who self monitored their blood glucose (SMBG) was small, there was no difference between DSAS‐1 scores of those using CGM compared to those who use SMBG. This could be because CGM is now the standard of care, so there is less variability of glucose monitoring modality within the T1D community. Alternatively, it could suggest that the device's visibility may not be the sole explanation for why insulin pump users reported elevated T1D‐stigma scores. Indeed, although not necessarily visible to others, individuals living with T1D describe how the pump increases their self‐consciousness, with one study reporting that participants who conceal their pumps feel that others can see right through them to the pump and diabetes.^31^, ^34^ A qualitative review provides evidence that the relationship between insulin pump use and stigma is complex.^34^ In particular, some users report that the pump is stigmatising, acting as a reminder of their diagnosis and label as an ‘outsider’. In contrast, others viewed it as normalising: an addition to the body that helps them to feel healthy.^35^ Future research would be helpful to further understand the relationship between diabetes technologies and T1D‐stigma.

Higher BMI was also correlated with higher T1D‐stigma scores. This may reflect broader obesity stigma, intersecting with misattributed type 2 diabetes stigma, compounding feelings of blame amongst people living with T1D.^7^ This is one example of the complex nature of T1D‐stigma and speaks to the international consensus recommendations for addressing diabetes stigma, which note a need for multi‐faceted solutions.^3^

Finally, the association between higher DSAS‐1 scores and self‐reported symptoms of anxiety, depression and a greater daily impact of diabetes supports the well‐evidenced broader emotional burden of T1D‐stigma.^1^, ^15^ However, it is important to note that there is likely a cyclical relationship between stigma and psychological distress: stigma may exacerbate mental health symptoms, which in turn can intensify the subjective experience of stigma and complicate diabetes self management.^1^, ^15^ Further qualitative exploration is necessary to understand the impact of T1D‐stigma.

There were a number of important diabetes outcomes: HbA1C, time in range, hypoglycaemia, diabetes course attendance and attendance for retinopathy or foot check, which were not associated with stigma scores in this study. Contrary to prior literature on adults^12^ and adolescents,^36^ there was no relationship detected between HbA1c and diabetes stigma scores in this study. An Australian study also found no clinically meaningful relationship between HbA1c and stigma scores^14^ and proposed that this may be due to the cross‐sectional nature of the study, and suggested that prospective longitudinal research would be helpful to better understand the relationship between diabetes stigma and diabetes‐related complications. There are limited data that show a relationship between hypoglycaemic episodes and diabetes stigma scores in adults,^37^ but research with adolescents suggests that more reported episodes are associated with higher stigma scores.^38^

## 5 LIMITATIONS

In the absence of existing UK data, our survey was necessarily observational, with no intervention‐based hypothesis testing being undertaken. Primarily, the data allowed for cross‐sectional, between‐group comparisons and exploration of associations between recorded variables. Since these were hypothesis‐generating comparisons, there has been no correction made for the possibility of spurious significances due to multiple statistical testing. Moreover, the small numbers in some groupings mean that conclusions should be treated with caution. For example, the higher mean DSAS‐1 score for the minoritised group compared to the White group was significant at *p* < 0.001, but it can be calculated that, given the observed means and SD, and with 400 participants in the White group, a further 13 would have been needed in the minoritised group to bring the power of the comparison up to 0.9.

Further, it should be noted that our response rate was lower than in other national T1D‐stigma studies at 9% compared to 11.5% (*N* = 900/*N* = 8000 invited) in the original DSAS‐1 validation paper,^8^ and 52% in Denmark^12^ (*N* = 1594/*N* = 3053 invited). Studies with similar methodology conducted in Switzerland,^11^ Canada,^4^ Australia^14^ and the United States^13^ included people with type 2 diabetes, and the response rates are not reported separately. The relatively low response rate suggests that there were barriers to engaging in the study. One of those could be that the term ‘stigma’ may be challenging to relate to or identify with. A previous study avoided using the term in their participant‐facing study materials to minimise the risk of attracting participants with extreme negative experiences and avoided using the word stigma in interviews unless the participant used it to prevent confusing them with jargon.^39^

Similar to previous studies,^1^ and despite working with a community agency to diversify recruitment, the sample was relatively homogenous across demographic, social and diabetes domains, for example, with 91.8% of participants identifying as White, university educated and reporting overall close to target HbA1c. This is not representative of many people living with T1D and may limit the generalisability of the results. This bias was in part due to the initial ADDRESS‐2 cohort from which the study recruited. While we attempted to widen the study's reach via other recruitment methods, this was unfortunately not as successful as we had hoped, perhaps due to language limitations or a need for a more active recruitment approach in minoritised ethnicity groups. Future studies may need to consider alternative routes and partnerships to increase participation.

## 6 CONCLUSION

These findings suggest that many adults with T1D in the UK report encountering T1D‐stigma, particularly those using insulin pumps, younger adults, women and individuals with higher BMI or who are experiencing mental health challenges.

It would be valuable for future research to qualitatively examine what contributes to people's experiences of T1D‐stigma in the United Kingdom to better understand how to tailor psychosocial support strategies that address and alleviate the impact of stigma for people with T1D.

In addition, longitudinal cohort studies may aid our understanding of why particular groups are more likely to encounter higher levels of perceived or experienced T1D‐stigma and how to mitigate its negative effects.

## CONFLICT OF INTEREST STATEMENT

None to declare.

## Data Availability

The data that support the findings of this study are available from the corresponding author upon reasonable request.

## APPENDIX A DSAS‐1 item‐response frequencies *N* and %

**Table:**

Subscale and item wording	Response options (*N* [%])				
	Strongly disagree	Disagree	Unsure	Agree	Strongly agree
Treated differently					
Because I have type 1 diabetes, I have been excluded by others from certain social events	159 (36)	173 (40)	49 (11)	47 (11)	10 (2.3)
Some people expect less of me because I have type 1 diabetes	62 (14)	138 (32)	121 (28)	104 (24)	13 (3.0)
I have been discriminated against in the workplace because I have type 1 diabetes	121 (28)	140 (32)	68 (16)	61 (14)	48 (11)
Some people think I'm unreliable because I have type 1 diabetes	83 (19)	159 (36)	106 (24)	67 (15)	23 (5.3)
I have been rejected by others (e.g. friends, colleagues, romantic partners) because of my type 1 diabetes	160 (37)	172 (39)	39 (8.9)	48 (11)	19 (4.3)
Some people see me as a lesser person because I have type 1 diabetes	79 (18)	124 (28)	120 (27)	97 (22)	18 (4.1)
Blame and Judgement					
Some people make unfair assumptions about what I can and cannot do because of my type 1 diabetes	25 (5.7)	52 (12)	26 (5.9)	216 (49)	119 (27)
Some people think I need insulin because I haven't looked after myself	36 (8.2)	79 (18)	75 (17)	181 (41)	67 (15)
Because I have type 1 diabetes, some people judge me if I eat sugary food or drinks (e.g., cakes, lollies, soft drink)	21 (4.8)	48 (11)	52 (12)	182 (42)	135 (31)
Some people think I'm irresponsible when my diabetes management isn't ‘perfect’	35 (8.0)	83 (19)	66 (15)	177 (40)	77 (18)
Some people assume that it is my fault I have type 1 diabetes (e.g., I ate too much sugar, I could have prevented it)	31 (7.1)	45 (10)	63 (14)	195 (45)	104 (24)
Some people think that I brought type 1 diabetes on myself	41 (9)	60 (14)	90 (21)	175 (40)	72 (16)
Identity concerns					
I feel embarrassed about what people might think if I need help with a hypo	40 (9.1)	93 (21)	45 (10)	159 (36)	101 (23)
I feel worried about telling people I have type 1 diabetes in case they react negatively	82 (19)	154 (35)	57 (13)	111 (25)	34 (7.8)
I feel self‐conscious about all the tools I need to manage my type 1 diabetes (e.g., insulin pen, pump, blood glucose meter)	78 (18)	135 (31)	27 (6.2)	139 (32)	59 (14)
To avoid negative reactions, I don't tell people I have type 1 diabetes	93 (21)	181 (41)	28 (6.4)	101 (23)	35 (8.0)
I feel embarrassed when I have to manage my type 1 diabetes in public (e.g., check blood glucose, inject/bolus insulin, refuse food, eat extra food)	72 (16)	141 (32)	29 (6.6)	150 (34)	46 (11)
If I were to inject insulin in public, people would think I was taking drugs	24 (5.5)	83 (19)	133 (30)	156 (36)	42 (9.6)
I worry what people will think if they see me injecting/bolusing insulin, or checking my blood glucose in public	69 (16)	137 (31)	31 (7.1)	148 (34)	53 (12)

*Note*: Data presented are *n* (%). Within rows, percentages do not always total 100 because of rounding.
